# Surgical treatment for insular gliomas. A systematic review and meta-analysis on behalf of the EANS neuro-oncology section

**DOI:** 10.1016/j.bas.2024.102828

**Published:** 2024-05-15

**Authors:** Matthias Simon, Anne Hagemann, Sanjana Gajadin, Francesco Signorelli, Arnaud J.P.E. Vincent, for the EANS Neuro-oncology Section

**Affiliations:** a Dept. of Neurosurgery, Bethel Clinic, University of Bielefeld Medical Center OWL, Bielefeld, Germany; b Society for Epilepsy Research, Bielefeld, Germany; c Department of Neurosurgery, Erasmus Medical Center, Rotterdam, the Netherlands; d Division of Neurosurgery, Department of Basic Medical Sciences, Neurosciences and Sense Organs, University "Aldo Moro", Bari, Italy

**Keywords:** Insular glioma, Extent of resection, Epilepsy, Monitoring/mapping, Awake craniotomy

## Abstract

**Introduction:**

The appropriate surgical management of insular gliomas is controversial. Management strategies vary considerably between centers.

**Research question:**

To provide robust resection, functional and epilepsy outcome figures, study growth patterns and tumor classification paradigms, analyze surgical approaches, mapping/monitoring strategies, surgery for insular glioblastoma, as well as molecular findings, and to identify open questions for future research.

**Material and methods:**

On behalf of the EANS Neuro-oncology Section we performed a systematic review and meta-analysis (using a random-effects model) of the more current (2000–2023) literature in accordance with the PRISMA guidelines.

**Results:**

The pooled postoperative motor and speech deficit rates were 6.8% and 3.6%. There was a 79.6% chance for postoperative epilepsy control. The postoperative KPI was 80–100 in 83.5% of cases. Functional monitoring/mapping paradigms (which may include awake craniotomies) seem mandatory. (Additional) awake surgery may result in slightly better functional but also worse resection outcomes. Transcortical approaches may carry a lesser rate of (motor) deficits than transsylvian surgeries.

**Discussion and conclusions:**

This paper provides an inclusive overview and analysis of current surgical management of insular gliomas. Risks and complication rates in experienced centers do not necessarily compare unfavorably with the results of routine neuro-oncological procedures. Limitations of the current literature prominently include a lack of standardized outcome reporting. Questions and issues that warrant more attention include surgery for insular glioblastomas and how to classify the various growth patterns of insular gliomas.

## Highlights

• Pooled 6.8%/3.6% motor/language deficit rates, and 79.6% epilepsy control.
• Functional monitoring/mapping paradigms (including awake craniotomies) mandatory.
• Possibly lesser rate of deficits with transcortical vs. transsylvian surgery.
• Surgery for insular glioblastomas and classification issues need more attention.

## Abbreviations

CNS: central nervous system
DFFITS: difference in fits
EANS: European Association of Neurosurgical Societies
EOR: extent of resection
GLMM: generalized linear mixed models
GBM: glioblastoma
GTR: gross total resection
IDH: isocitrate (1) dehydrogenase
ILAE: International League Against Epilepsy
KPI: Karnofsky performance index
LGG: low grade (=WHO/CNS grade 2) gliomas
LSA: lenticulo-striate arteries
mNOS: modified Newcastle-Ottawa scale
MR(I): magnetic resonance (imaging)
NA: not assessed/applicable
OR: odds ratio
PRISMA: Preferred Reporting Items for Systematic Reviews and Meta-Analyses
WHO: World Health Organization
95% CI: 95% confidence interval

## 1 Introduction

Surgical management of gliomas involving the insula poses significant challenges. There is a growing body of evidence that the degree of glioma resection is an important prognostic parameter, i.e. cytoreductive surgery for gliomas likely improves patient survival (Gousias et al., 2014; Hervey-Jumper et al., 2023; Stummer et al., 2006). However, aggressive surgery may come with a higher rate of neurological deficits which has detrimental effects not only on functional but also oncological outcomes (Gulati et al., 2011). Insular surgery has traditionally been associated with very substantial neurological risks probably reflecting the location and in particular the vascularization of this part of the brain. Hence, treatment of such patients is challenging, difficult and often controversial.

Following the initial and seminal publications by Yasargil and co-workers (Yaşargil et al., 1992) many institutions have offered surgical treatment for insular gliomas. The pertinent literature consists largely of case series reporting the institutional experience of the respective authors usually over a longer time period. The definition and classification of the tumors as well as surgical approaches and use of technical adjuncts vary between institutions. Publications typically do not detail control groups and/or describe patients not undergoing resective surgery.

The panel of the EANS Neuro-oncology Section decided to address selected controversies in surgical neuro-oncology by performing systematic reviews of the pertinent literature and current practices. Surgical management of insular gliomas was identified as an appropriate topic. As pointed out above, the literature on insular glioma surgery is not unsubstantial. The present paper provides a systematic review and meta-analysis of the published experience and attempts to identify specific questions which may deserve attention by researchers in the future.

## 2 Methods and materials

### 2.1 Task force and identification of research questions

The panel of the EANS Neuro-oncology Section (https://www.eans.org/page/neuroncology-section) selected three individuals (FS, MS, AV) in a consensual manner in order to conduct a systematic review of insular glioma surgery. This task force conducted a formal discussion aiming at the identification of areas of interest and open questions in a consensual manner. The following questions and issues of interest were identified.

1. Resection as well as
2. Functional and
3. Epilepsy outcomes.
4. Growth pattern and tumor classification.
5. Surgical approach and
6. Mapping/monitoring strategies.
7. Surgery for insular glioblastoma.
8. Molecular genetic findings.

### 2.2 Literature search and study selection

In compliance with the PRISMA guidelines, we systematically searched the Embase, Medline ALL, Web of Science Core Collection, Cochrane Central Register of Controlled Trials and Google Scholar databases for relevant publications describing surgical management of insular gliomas (Suppl. Table 1). All studies published before June 2023 were screened. Articles published before 2000, describing less than 10 cases or which did not provide data on mortality, morbidity, or tumor resection, were excluded. We also excluded articles published in another language than English, articles not available in full text, and editorials. All articles had to be original and peer reviewed. If several publications were identified describing an accumulating patient cohort, only the most recent paper detailing the required data was included. Study eligibility was assessed manually by three authors (FS, MS and AV). Differences were resolved by majority voting.

**Fig. 1:**
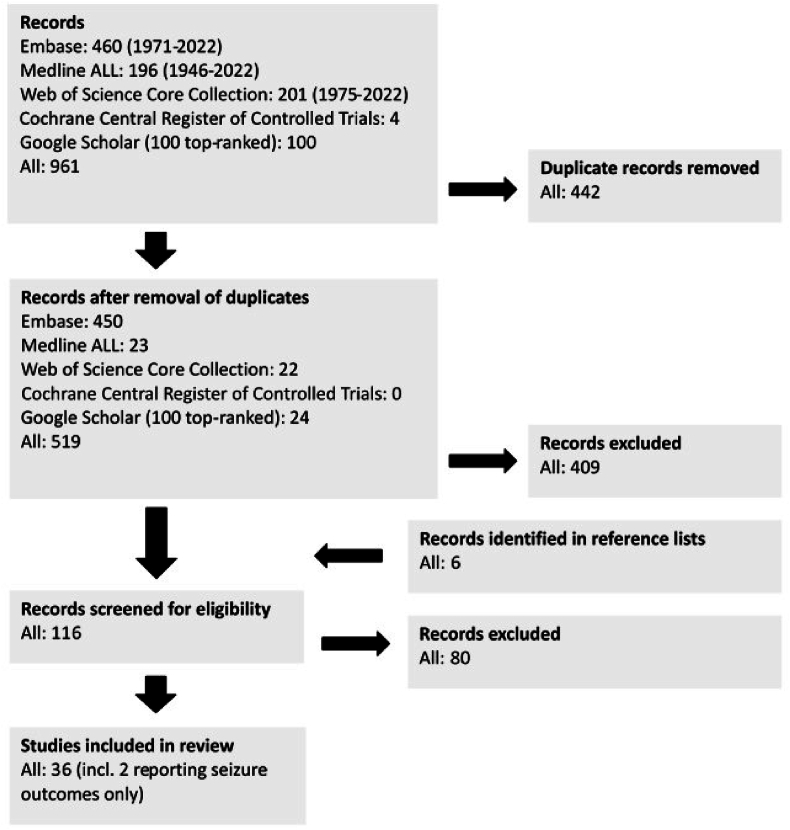
Search and selection strategy (PRISMA flow diagram).

We included 36 studies in the final investigation. Two papers investigated epilepsy outcomes only and were analyzed only with respect to freedom of seizures (Ius et al., 2014; Wang et al., 2018). All other analyses were performed with the remaining 34 studies (Fig. 1). Individual case data were not available for the larger patient cohorts which prompted us to collect and analyze rates reported in the respective series rather than individual case data. Preoperative clinical signs and symptoms and postoperative neurological and epilepsy outcomes were not reported in a standardized fashion. Based on the data found in the majority of the articles reviewed for each item we documented pre- and postoperative motor and speech deficit rates, percentage of cases presenting with epilepsy, rate of epileptic patients reported as “seizure-free” following surgery, and percentage of patients with a KPI of 80–100 after surgery. Robust information with respect to the severity of deficits, or non-motor – non-language/overall rates of deficits could not be retrieved from the great majority of papers. We therefore documented any postoperative motor and language deficits. If information for various time points was available, we documented the >3 months figures. Many publications also distinguished between early and late/permanent deficits, however, the time-points at which early deficits were assessed varied widely between <24 h And 2 weeks. Because of this variation and since temporary deficits often improve rapidly, we elected not to study temporary deficits in detail. Information on the use of mapping and monitoring strategies including awake surgeries was also collected as available.

Extent of resection is also not described in a standardized manner in the literature and different definitions of the “best” resection category were used. We therefore recorded extent of resection as the percentage of cases reported with a >90% (95%) resection or a gross total or total/complete resection. Volumetric data was rarely provided and therefore not analyzed further. Histological findings were categorized as “glioblastoma”/WHO or CNS grade 4, “anaplastic glioma”/grade 3, “low grade glioma”/grade 2, grade 1 tumors and “other” based on the information detailed in the majority of publications. Of note, the majority of the studies were published before the 2021 revision of the WHO classifications and therefore do not use molecular findings such as IDH mutations and 1p/19q co-deletions to define the various histological categories (Louis et al., 2021). Survival was estimated from the Kaplan Meier curves shown in the respective manuscript if not specifically mentioned in the text. For comparison we attempted to list 1 year overall survival for glioblastomas/WHO or CNS grade 4 tumors, and 5 year overall survival for anaplastic and “low grade”/grade 2 tumors.

### 2.3 Quality assessment and risk of bias

We assessed the quality of the studies reviewed using a modified Newcastle Ottawa Scale (mNOS) for cohort studies (Stang, 2010). Similar to others (Roa Montes de Oca et al., 2022) we evaluated only the sample selection and outcome reporting domains allowing for a maximum of 6 points. Representativeness was assessed based on the spectrum of pathologies (inclusion of WHO grade II-IV cases), clinical presentations and growth patterns (inclusion of cases with extrainsular growth) reported and a description of the imaging and the monitoring/mapping paradigms employed. Positive assessments of outcome reporting required detailing motor and speech deficit rates, KPI, epilepsy and extent of resection outcomes. We are aware that this evaluation does not measure the scientific quality of the respective papers but rather the usefulness of the data reported for the purposes of this review.

The median mNOS score was 5 (95% CI: 4–6, range: 4–6). Publication bias risk was studied by examining funnel plots for asymmetry using regression testing.

### 2.4 Meta-analysis

We used meta-analytical models to combine the proportions extracted from the individual studies into a pooled estimate. Assuming a random-effects model, we used generalized linear mixed models (GLMM) with logit-link to estimate the pooled proportions and the corresponding 95% confidence intervals (95% CI) in a single step (Lin and Chu, 2020). Heterogeneity was assessed with the I^2^ statistic and Q tests, with I^2^-values of 25%., 50%, and 75% corresponding to low, medium, and high heterogeneity. Outliers and influential cases were identified with diagnostic measures (e.g., studentized residuals, DFFITS, Cook's distance). If such cases were identified, the analysis was repeated after their exclusion and results were compared. Forest plots were created to illustrate the proportions and confidence intervals from the individual studies and the pooled estimate. For subgroup analyses (e.g., surgical approach), the respective grouping variable was included as a categorical moderator and χ^2^-tests were used to test for subgroup differences. To compare the functional outcomes of insular glioblastomas with that of other histologies, pooled odds ratios (OR) were estimated for those studies providing information on both groups. We used the R software (version 4.3.0, packages “meta” and “metafor”) for statistical analyses.

## 3 Results

### 3.1 Studies & patient cohorts

We identified a total of 34 publications describing 2231 patients meeting our inclusion criteria (Table 1a, Table 1ba and 1b). Two further papers (Ius et al., 2014; Wang et al., 2018) detailed epilepsy outcomes only while other pertinent data of the respective institutional cohorts could be found in separate publications (Hervey-Jumper et al., 2016; Sanai et al., 2010; Skrap et al., 2012). Patients presented with motor deficits in 8.0% (pooled proportion; 95% CI: 4.5%–12.7%), speech deficits in 5.8% (95% CI: 3.6%–9.2%) and epilepsy in 66.5% (95% CI: 58.9%–73.3%; Table 2). Eight studies describe patients (N = 33) with glioneuronal tumors, CNS/WHO grade 1 gliomas or non-glial tumors (Kawaguchi et al., 2014; Lang et al., 2001; Majchrzak et al., 2011; Moshel et al., 2008; Özyurt et al., 2003; Pitskhelauri et al., 2021; Rao et al., 2018; Simon et al., 2009) (Table 1A).

**Table 1a:** Case series included in the present analysis: clinical presentation, histologies, clinical outcomes and survival.

	Cases	Clinical presentation (per surgical case)	Histology	Outcomes				
	N			Postop. Motor deficit	Postop. speech deficit	Functional outcomes	Seizure outcomes (seizure-free/epilepsy at presentation)	Overall survival by histology
Lang et al. (2001)	22	Epilepsy: 14 (63.6%), motor deficit: 7 (31.8%), dysphasia: 4 (18.2%)	GBM: 5 (22.7%), anapl.: 6 (27.3%), LGG: 10 (45.5%), WHO°I: 0, other: 1 (4.5%)	2 (9.1%)	0	NA	NA	NA
Özyurt et al. (2003)	40	Epilepsy: 25 (62.5%), motor deficit: 9 (22.5%), dysphasia: 0, incidental: 3 (7.5%)	GBM: 7 (17.5%), anapl.: 6 (15.0%), LGG: 22 (55.0%), WHO°I: 3 (7.5%), other: 2 (5.0%)	2 (5.0%)	1 (2.5%)	NA	21/25 (84%)	NA
Moshel et al. (2008)	38	Epilepsy: 27 (71.1%), motor deficit: 3 (7.9%), dysphasia: 6 (15.8%)	GBM: 4 (10.5%), anapl.: 6 (15.8%), LGG: 16 (42.1%), WHO°I: 11 (28.9%), other: 1 (2.6%)	5 (13.2%)	2 (5.3%)	NA	NA	NA
Duffau (2009)	51	Epilepsy: 50 (98.0%), intracranial hypertension: 1 (2.0%)	GBM: 0, anapl.: 0, LGG: 51 (100%), WHO°I: 0, other: 0	2 (3.9%)	0	KPI 80–100: 49 (96.1%), ≤70: 2 (3.9%)	14/18 (77.7%)	LGG: 76% 5 yrs.
Simon et al. (2009)	101 (94 patients)	Epilepsy: 83 (82.2%), motor deficit and/or dysphasia: 24 (23.8%)	GBM: 21 (20.8%), anapl.: 44 (43.6%), LGG: 30 (29.7%), WHO°I: 6 (5.9%), other: 0	12/96 (12.5%)	5/96 (5.2%)	KPI 80–100: 68/100 (68.0%), ≤70: 32/100 (32.0%)	42/55 (76%)	GBM: 50% 1 yr., anapl.: 63.5% 5 yrs., LGG: 68% 5 yrs.
Sanai et al. (2010)	115 (104 patients)	Epilepsy: 75 (65.2%), motor deficit: 0, dysphasia: 5 (4.3%)	GBM: 10 (8.7%), anapl.: 35 (30.4%), LGG: 70 (60.9%), other: 0	5 (4.3%)	1 (0.9%)	NA	NA	GBM: 68% 1 yr., anapl.: 74% 5 yrs., LGG: 77% 5 yrs.
Majchrzak et al. (2011)	30	Generalized seizures: 7 (23.3%), partial seizures: 26 (86.6%), motor deficit: 2 (6.6%), dysphasia: 0	GBM: 3 (10.0%), anapl.: 9 (30.0%), LGG: 16 (53.3%), WHO°I: 1 (3.3%), other: 1 (3.3%)	4 (13.3%)	4 (13.3%)	KPI 80–100: 22 (73.3%), ≤70: 8 (26.7%)	NA	NA
Skrap et al. (2012)	71 (66 patients)	Epilepsy: 64 (90.1%), motor deficit: 1 (1.4%), dysphasia: 3 (4.2%), intracranial hypertension: 2 (2.8%)	GBM: 0, anapl.: 13/66 (18.3%), LGG: 53/66 (80.3%), WHO°I: 0, other: 0	2/66 (3.0%)	2/66 (3.0%)	NA	NA	anapl.: 58% 5 yrs., LGG: 85% 5 yrs.
Wang et al. (2012)	12	Epilepsy: 8 (66.7%), motor deficit: 2 (16.7%), dysphasia: 0	NA	4 (33.3%)	2 (16.7%)	NA	NA	NA
Kawaguchi et al. (2014)	83	NA	GBM: 31 (37.3%), anapl.: 33 (39.8%), LGG: 14 (16.9%), WHO°I: 5 (6.0%), other: 0	17 (20.5%)	9 (10.8%)	NA	NA	GBM: 82% 1 yr., anapl.: 75% 5 yrs., LGG: 100% 5 yrs.
Ius et al. (2014) (overlap with Skrap et al., 2012)	52	Epilepsy: 52 (100%), motor deficit & dysphasia: NA	GBM: 0, anapl.: 0, LGG: 52 (100%), WHO°I: 0, other: 0	NA	NA	NA	35 (67.3%)	NA
Alimohamadi et al. (2016)	10	Epilepsy: 8 (80.0%), motor deficit: 1 (10.0%), dysphasia: 3 (30.0%)	GBM & anapl.: 3 (30.0%), LGG: 7 (70.0%), WHO°I: 0, other: 0	0	1 (10.0%)	NA	1/4 (25.0%; med. Refractory seizures)	NA
Barbosa et al. (2016)	28	Epilepsy: 15 (53.6%), focal deficit: 9 (32.1%), intracranial hypertension: 5 (17.9%)	GBM & anapl.: 20 (71.4%), LGG: 8 (28.6%)	NA	NA	NA	NA	HGG: 70% 1 yrs., 5 yrs. NA
Hervey-Jumper et al. (2016)	129 (114 patients)	Epilepsy: 88 (68.2%), motor deficit: 3 (2.3%), dysphasia: 1 (0.8%)	GBM: 15 (11.6%), anapl.: 44 (34.1%), LGG: 70 (54.3%), WHO°I: 0, other: 0	2 (1.6%)	1 (0.8%)	NA	NA	NA
Sughrue et al. (2016)	20	NA	GBM: 5 (25.0%), anapl.: 6 (30.0%), LGG: 9 (45.0%), WHO°I: 0, other: 0	2 (10.0%)	0 (0%)	KPI 80–100: 14 (70.0%), ≤70: 6 (30.0%)	NA	NA
Tang et al. (2016)	42	NA	LGG: 42 (100%)	NA	NA	NA	NA	LGG: 68% 5 yrs.
Zhuang et al. (2016)	30	Epilepsy: 18 (60.0%), sensorimotor deficit: 3 (10.0%), dysphasia: 5 (50.0%)	GBM: 6 (20.0%), anapl.: 3 (10.0%), LGG: 21 (70.0%), WHO°I: 0, other: 0	4 (13.3%)	5 (16.7%)	NA	11/18 (61.1%)	NA
Eseonu et al. (2017)	74	Epilepsy: 26 (35.1%), motor deficit 17 (23.0%), dysphasia: 12 (16.2%)	GBM: 33 (44.6%), anapl.: 16 (21.6%), LGG: 25 (33.8%), WHO°I: 0, other: 0	2 (2.7%)	5 (6.8%)	NA	NA	GBM: 96% 1 yr., anapl.: 62% 5 yrs., LGG: 93% 5 yrs.
Chen et al. (2017)	73	Epilepsy: 50 (68.5%), motor deficit 9 (12.3%), dysphasia: 5 (6.8%)	GBM: 62 (84.9%), anapl.: 11 (15.1%)	6 (8.2%)	0	NA	49/50 (98.0%)	NA
Sughrue et al. (2017)	72	Epilepsy: NA, motor deficit: 23 (31.9%), dysphasia: 11 (15.3%)	GBM: 33 (45.8%), anapl.: 16 (22.2%), LGG: 23 (31.9%), WHO°I: 0, other: 0	12 (16.7%)	3 (4.2%)	KPI 80–100: 58/68 (85.0%), ≤70: 10/68 (15.0%)	NA	GBM: 55% 1 yr., anapl.: 83% 5 yrs., LGG: 35% 5 yrs.
Wang et al. (2017)	211	Epilepsy: 120 (56.9%), motor deficit & dysphasia: NA	GBM: 0, anapl.: 0, LGG: 211 (100%), WHO°I: 0, other: 0	NA	NA	NA	NA	NA
Wang et al. (2017) (overlap with Hervey-Jumper et al., 2016)	109	Epilepsy: 109 (100%), motor deficit & dysphasia: NA	GBM: 7 (6.4%), anapl.: 36 (33.0%), LGG: 66 (60.6%), WHO°I: 0, other: 0	NA	NA	NA	74 (67.9%)	NA
Baran et al. (2018)	22	Epilepsy: 19 (86.4%), motor deficit: 5 (22.7%), dysphasia: 0	GBM: 1 (4.5%), anapl.: 3 (13.6%), LGG: 18 (81.8%), WHO°I: 0, other: 0	1 (4.5%)	1 (4.5%)	NA	17/19 (89.5%)	NA
Rao et al. (2018)	48	NA	GBM: 3 (6.3%), anapl.: 29 (60.4%), LGG: 15 (31.3%), WHO°I: 1 (2.1%), other: 0	6 (12.5%)	NA	NA	NA	NA
Hameed et al. (2019)	255	Epilepsy: 105 (41.2%), motor deficit & dysphasia: NA	GBM: 81 (31.8%), anapl.: 54 (21.2%), LGG: 120 (47.1%), WHO°I: 0, other: 0	21/172 (12.2%)	12/172 (7.0%)	NA	NA	IDHwt GBM: 64% 1 yr., IDHmut anapl. & LGG: 57% 5 yrs.
Khatri et al. (2020)	41	Epilepsy: 24 (58.5%), motor deficit: 17 (41.5%), dysphasia: 1 (2.4%), intracranial hypertension (papilledema): 19 (46.3%)	GBM: 21 (51.2%), anapl.: 20 (48.8%), LGG: 0, WHO°I: 0, other: 0	3 (7.3%)	1 (2.4%)	NA	NA	GBM: 24% 1 yr., anapl.: 53% 5 yrs.
Li et al. (2020)	253	Epilepsy: 144 (57.1%), motor deficit: 27 (10.7%), dysphasia 8 (3.2%).	GBM: 30 (11.9%), anapl.: 74 (29.2%), LGG: 149 (58.9%), WHO°I: 0, other: 0	5 (2.0%)	3 (1.2%)	NA	NA	NA
Przybylowski et al. (2020)	100	Epilepsy: 61 (61.0%), motor deficit & dysphasia: NA	GBM & anapl.: 68 (68.0%), LGG: 32 (32.0%)	10 (10.0%)	3 (3.0%)	NA	NA	NA
Leroy et al. (2021)	20	Epilepsy: 14 (70.0%), motor deficit & dysphasia: 0 (excluding neuropsychological testing)	GBM: 4 (20.0%), anapl.: 4 (20.0%), LGG: 12 (60.0%), WHO°I: 0, other: 0	1 (5.0%)	0	KPI 80–100: 19 (95.0%), ≤70: 1 (5.0%)	NA	NA
Pallud et al. (2021)	149 (111 resection & 38 biopsy cases)	Epilepsy: 111 (74.5%), focal deficit: 29 (19.5%)	GBM: 30 (20.1%), anapl.: 53 (35.6%), LGG: 66 (44.3%), WHO°I: 0, other: 0	3/61 (resective cases, 4.9%)	NA	NA	56/68 (82.4%)	IDHwt GBM: 39% 1 yr., anapl. & IDHmut LGG: 67% 5 yrs.
Panigrahi et al. (2021)	61	Epilepsy: 34 (55.7%), motor deficit: 7 (11.5%), dysphasia: 7 (11.5%), intracranial hypertension: 11 (18.0%)	GBM: 12 (19.7%), anapl.: 7 (11.5%), LGG: 42 (65.6%), WHO°I: 0, other: 0	4 (6.6%)	4 (6.6%)	NA	NA	NA
Pepper et al. (2021)	38	Epilepsy: 9 (23.7%), motor deficit: 0, dysphasia: 3 (7.9%), intracranial hypertension: 2 (5.3%)	GBM: 12 (31.6%), anapl.: 26 (68.4%), LGG: 0, WHO°I: 0, other: 0	4 (10.5%)	2 (5.3%)	NA	NA	NA
Pitskhelauri et al. (2021)	79	Epilepsy: 58 (73.4%), motor deficit: 3 (3.8%), dysphasia: 4 (5.1%), intracranial hypertension: 1 (1.3%)	GBM: 15 (19.0%), anapl.: 11 (13.9%), LGG: 52 (65.8%), WHO°I: 1 (1.3%), other: 0	4 (5.1%)	1 (1.3%)	NA	NA	NA
Rossi et al. (2021)	95	Epilepsy: 70 (73.7%), motor deficit: 11 (11.6%), dysphasia 16 (16.8%)	GBM: 19 (20.0%), anapl.: 7 (7.4%), LGG: 69 (72.6%), WHO°I: 0, other: 0	7 (7.4%)	20 (21.1%)	NA	38/43 (uncontrolled seizures, 88.4%)	NA
Sun et al. (2022)	69	Epilepsy: 38 (55.1%), motor deficit: 7 (10.1%), dysphasia: 7 (10.1%)	GBM & anapl.: 29 (42.0%), LGG: 40 (58.0%)	NA	1 (1.4%)	NA	NA	NA
Singh et al. (2023)	27	Epilepsy: 14 (51.9%), focal deficit: 9 (33.3%), intracranial hypertension: 14 (51.9%)	GBM: 27 (100%), anapl.: 0, LGG: 0, WHO°I: 0, other: 0	NA	NA	NA	NA	GBM: 0% 1 yr.

NA: not assessed/applicable.

GBM: glioblastoma.

anapl.: anaplastic (=WHO/CNS grade 3) gliomas.

LGG: low grade (=WHO/CNS grade 2) gliomas.

WHO°I: WHO/CNS grade 1 tumors (i.e. pilocytic astrocytomas, glio-neuronal tumors).

other: all other histologies.

KPI: Karnofsky performance index.

yr(s).: year(s).

IDHwt/mut: isocitrate 1 dehydrogenase wild-type/mutant.

**Table 1b:** Case series included in the present analysis: growth pattern, surgical approaches, monitoring/mapping & imaging paradigms, and extent of resection outcomes.

	Cases	Growth pattern		Surgical approach		Surgical adjuncts, imaging	Extent of resection
	N	Extrainsular growth (per surgical case)	Classification scheme used	(if available: N, %)	Transsylvian approach N (%)	Modalities (if available): N (%)	EOR >90%^a^
Lang et al. (2001)	22	14 (63.6%)	NA	TS (8, 36.4%), TS + TT (8, 36.4%), TS + TOP (6, 27.3%)	22 (100%)	Neuronavigation + US: 22 (100%), awake: 5 (22.7%), iopM (SEPs), electrostim.	10 (45.5%)
Özyurt et al. (2003)	40	33 (82.5%)	(Yasargil); types 1 to 4 correspond to Yasargil types 3 A, 3 B, 5 A and 5 B	TS (40, 100%)	40 (100%)	NA	24 (60.0%)
Moshel et al. (2008)	38	20 (52.6%)	Diffuse vs. sharp medial border & LSA involvement	TS ± TT or TF (38, 100%)	38 (100%)	iopM (MEPs&SEPs), electrostim.: 38 (100%)	31 (81.6%)
Duffau (2009)	51	44 (86.3%)	Yasargil	TS (3, 5.9%), TOP (48, 94.1%)	3 (5.9%)	electrostim.: 35 (70.0%), awake: 16 (31.4%), US, iopM	8 (15.7%)
Simon et al. (2009)	101 (94 patients)	91 (90.0%)	Yasargil	TS ± TT/TF (51, 50.5%), TT or TF (50, 49.5%)	51 (50.5%)	Neuronavigation, iopM (MEPs&SEPs)	42/100 (42.0%)
Sanai et al. (2010)	115 (104 patients)	NA	Berger-Sanai	TOP (115, 100%)	0	Electrostim.: 50 (43.5%), awake: 65 (56.5%)	24/104 (23.1%)
Majchrzak et al. (2011)	30	21 (70.0%)	Yasargil	TS ± TT or TF (30, 100%)	30 (100%)	Neuronavigation: 21 (70.0%), fMRI, iopM (MEPs), electrostim.: 30 (100%)	16 (53.3%)
Skrap et al. (2012)	71 (66 patients)	64/66 (97.0%)	Yasargil	TS,TF,TT	NA	Neuronavigation, fMRI, iopM (MEPs&SEPs): 71 (100%), electrostim.: 28 (39.4%), awake: 43 (60.6%)	22/66 (33.3%)
Wang et al. (2012)	12	9 (75.0%)	(Yasargil); types I to IV correspond to Yasargil types 3 A, 3 B, 5 B and 5 A	TS (12, 100%)	12 (100%)	DTI (12, 100%) US	9 (75.0%)
Kawaguchi et al. (2014)	83	NA	Kawaguchi classification	NA	NA	Awake: 5 (6.0%), MRmicroangio: 83 (100%), neuronavigation, iopM (MEPs&SEPs)	37 (44.6%)
Ius et al. (2014) (overlap with Skrap et al., 2012)	52	NA	NA	TS, TF, TT (cf. Skrap et al., 2012)	NA	Neuronavigation, iopM (MEPs&SEPs): 52 (100%), electrostim.: 16 (30.8%), awake: 36 (69.2)	21 (40.4%)
Alimohamadi et al. (2016)	10	NA	NA	NA	NA	Neuronavigation, SPECT, fMRI, DTI: 10 (100%), iopM (MEPs), electrostim.	5 (50.0%)
Barbosa et al. (2016)	28	20 (74.1%)	NA	NA	NA	Neuronavigation: 19 (67.9%), DTI: 8 (28.6%), iopM (MEPs&SEPs): 18 (64.3%)	6/27 (22.2%)
Hervey-Jumper et al. (2016)	129 (114 patients)	NA	Berger-Sanai	TOP (129, 100%)	0	electrostim.: 71 (55.0%), awake: 58 (45.0%)	51/129 (39.5%)
Sughrue et al. (2016)	20	0	NA	TS (20, 100%)	20 (100%)	Neuronavigation (20, 100%), endoscopy	18 (90.0%)
Tang et al. (2016)	42	22 (52.4%)	NA	NA	NA	NA	19 (54.8%)
Zhuang et al. (2016)	30	17 (56.7%)	Berger-Sanai	TS (7, 23.3%), TOP (17, 56.7%), TS + TOP (6, 20.0%)	13 (43.3%)	Neuronavigation, DTI, iopMRI, iopM (MEPs): 30 (100%), electrostim.: 10 (33.3%), awake: 20 (66.7%)	23 (40.8%)
Eseonu et al. (2017)	74	NA	Berger-Sanai	TOP (74, 100%)	0	Electrostim.: 45 (60.8%), awake: 29 (39.2%)	40 (54.1%)
Chen et al. (2017)	73	NA	Berger-Sanai	TS (5, 6.8%), TOP, TF or TT (68, 93.2%)	5 (6.8%)	Neuronavigation: 73 (100%), iopMRI: 51 (69.9%), DTI	47 (64.4%)
Sughrue et al. (2017)	72	72 (100%)	NA	TS, TF, TT	NA	Neuronavigation: 72 (100%)	67 (93.1%)
Wang et al. (2017)	211	NA	Yasargil, Berger-Sanai, Putamen classification	NA	NA	NA	81 (38.4%)
Wang et al. (2017) (overlap with Hervey-Jumper et al., 2016)	109	NA	Berger-Sanai	TOP (109, 100% (cf. Sanai et al., 2010)	0	Electrostim.: 49 (45.0%), awake: 60 (55.0%)	33 (30.3%)
Baran et al. (2018)	22	18 (81.8%)	NA	TOP (22, 100%)	0	Neuronavigation: 22 (100%), awake: 11 (50.0%)	22 (100%)
Rao et al. (2018)	48	NA	NA, LSA encased vs. pushed	TS (48, 100%)	48 (100%)	US, awake	5 (22.7%)
Hameed et al. (2019)	255	203 (79.6%)	Berger-Sanai	TOP (255, 100%)	0	Neuronavigation, DTI, iopM (MEPs): 255 (100%), fMRI, iopMRI, electrostim., awake	172 (67.5%)
Khatri et al. (2020)	41	40 (97.6%)	Yasargil, Berger-Sanai	TS (13, 31.7%), TOP (28, 68.3%)	13 (31.7%)	Neuronavigation: 6 (14.6%), US: 4 (9.8%), iopM (MEPs), electrostim.: 5 (12.2%), awake: 2 (4.9%)	23 (56.1%)
Li et al. (2020)	253	NA	Berger-Sanai	TOP (253, 100%)	0	fMRI, iopM, electrostim.	176 (69.6%)
Przybylowski et al. (2020)	100	NA	Berger-Sanai	TS (52, 52.0%), TOP (48, 48.0%)	52 (52.0%)	Neuronavigation: 100 (100%), fMRI, DTI, electrostim., awake	(mean EOR, trans-sylvian vs. not: 91.6 vs. 88.6%)
Singh et al. (2023)	27	18 (33.3%)	Yasargil, Berger-Sanai	TS (13, 48.1%), TOP (14, 51.9%)	13 (48.1%)	Neuronavigation: 4 (14.8%), US: 4 (14.8%), iopM (MEPs): 3 (11.1%), electrostim.: 3 (11.1%)	16 (59.3%)
Leroy et al. (2021)	20	NA	Berger-Sanai	TOP, TT	0	Neuronavigation, fMRI, iopMRI: 20 (100%)	12 (60.0%)
Pallud et al. (2021)	149 (111 resection & 38 biopsy cases)	138/149 (92.6%)	Yasargil, Berger-Sanai	TOP (111, 100%)	0	Neuronavigation: 111/149 (74.5%), US, awake: 61/149 (40.9%)	21 (14.1%)
Panigrahi et al. (2021)	61	NA	Berger-Sanai	TS (38, 62.3%), TOP (23, 37.7%)	38 (62.3%)	Neuronavigation, fMRI, DTI, electrostim., ICG angio: 61 (100%)	38 (62.3%)
Pepper et al. (2021)	38	NA	Berger-Sanai	NA	NA	iopM: 11 (28.9%), awake: 27 (71.1%)	6 (15.8%)
Pitskhelauri et al. (2021)	79	38 (48.1%)	Berger-Sanai, Pitskhelauri classification	TS (77, 97.5%), TS + TOP (2, 2.5%)	79 (100%)	iopM (MEPs): 79 (100%), electrostim., awake: 2 (2.5%)	30 (38.0%)
Rossi et al. (2021)	95	81 (85.3%)	Berger-Sanai, diffuse vs. sharp medial border, LSA, deep perforator and opercular arteries involvement	TOP (95, 100%)	0	iopM (MEPs&SEPs), ECOG: 95 (100%), electrostim.: 25 (26.3%), awake: 70 (73.6%)	70 (73.7%)
Sun et al. (2022)	59	NA	Berger-Sanai	TF (59, 100%)	0	Neuronavigation, fMRI, iopMRI, iopM (MEPs): 59 (100%)	45 (76.3%)
Singh et al. (2023)	27	18 (33.3%)	Yasargil, Berger-Sanai	TS (13, 48.1%), TOP (14, 51.9%)	13 (48.1%)	Neuronavigation: 4 (14.8%), US: 4 (14.8%), iopM (MEPs): 3 (11.1%), electrostim.: 3 (11.1%)	16 (59.3%)

NA: not assessed/applicable.

LSA: lenticulo-striate arteries.

TS, TT, TF, TOP: trans-sylvian, transtemporal, transfrontal, transopercular approach.

US: intraoperative ultrasound.

MRI: magnetic resonance imaging.

fMRI: functional MRI.

DTI: diffusion tensor imaging.

MRmicroangio: MR microangiography (3 T 3D time-of flight MR angiography) (Kawaguchi et al., 2014).

iopM: intraoperative monitoring.

MEPs, SEPs: motor evoked, sensory evoked potentials.

electrostim.: general anesthesia & intraoperative (sub)cortical electrostimulation mapping.

awake: awake surgery ± intraoperative (sub)cortical electrostimulation mapping.

a Cases with a gross total resection or EOR (extent of resection) > 90 (95)%.

**Table 2:** Pooled weighted clinical and tumor characteristics and outcomes.

	n (studies)	n (cases)	Pooled proportion (95% CI)	Heterogeneity
Preoperative deficits				
Motor deficit	22	1410	8.0% (4.5%–12.7%)	I^2^ = 73.1% (59.0%–82.4%), p < 0.001
Dysphasia^a^	23	1440	5.8% (3.6%–9.2%)	I^2^ = 54.2% (26.5%–71.4%), p = 0.001
Preop epilepsy	28	2209	66.5% (58.9%–73.3%)	I^2^ = 83.8% (77.5%–88.3%), p < 0.001
Extrainsular growth^b^	19	1199	81.7% (72.2%–88.5%)	I^2^ = 84.7% (77.3%–89.6%), p < 0.001
EOR >90%^c^	33	2392	53.7% (43.6%–63.5%)	I^2^ = 91.2% (88.7%–93.1%), p < 0.001
Histology^d^				
GBM	29	2285	16.2% (8.7%–28.1%)	I^2^ = 86.3% (81.5%–89.9%), p < 0.001
Anaplastic	29	2285	17.9% (11.6%–26.5%)	I^2^ = 79.7% (71.5%–85.5%), p < 0.001
LGG	33	2492	48.9% (29.7%–68.5%)	I^2^ = 81.7% (75.0%–86.5%), p < 0.001
WHO°1^e^	33	2492	0.1% (0.0%–0.8%)	I^2^ = 0.0% (0.0%–39.2%), p = 0.792
Other^f^	33	2492	0.0% (0.0%–1.2%)	I^2^ = 0.0% (0.0%–39.2%), p > 0.999
Outcomes				
Postop. motor deficit^g^	29	1951	7.4% (5.6%–9.7%)	I^2^ = 56.6% (34.2%–71.3%), p < 0.001
Postop. speech deficit	29	1959	3.3% (2.1%–5.3%)	I^2^ = 58.4% (37.2%–72.4%), p < 0.001
Postop. KPI 80–100^h^	6	290	83.5% (70.7%–91.4%)	I^2^ = 73.4% (39.1%–88.4%), p = 0.002
Seizure-free^i^	11	461	79.6% (70.1%–86.7%)	I^2^ = 62.2% (27.3%–80.3%), p = 0.003

EOR: extent of resection.

95% CI: 95% confidence interval.

GBM: glioblastoma.

anapl.: anaplastic (=WHO/CNS grade 3) gliomas.

LGG: low grade (=WHO/CNS grade 2) gliomas.

WHO°I: WHO/CNS grade 1 tumors (i.e. pilocytic astrocytomas, glio-neuronal tumors).

other: all other histologies.

KPI: Karnofsky performance index.

a Li et al. (2020) was identified as an influential case (largest sample size) and regression test showed significant funnel plot asymmetry (p = .015). After exclusion of this study, the pooled proportion did not change considerably (6.0%, 95% CI: 3.6%–9.7%) and the regression test was no longer significant (p = .171).

b Exclusion of Sughrue et al. (2016) because patients with extrainsular growth were not included in this study.

c Cases with a gross total resection or EOR (extent of resection) > 90 (95)%.

d Three studies included only LGG (Duffau, 2009; Tang et al., 2016; Wang et al., 2017), one study included only GBM (Singh et al., 2023).

e Moshel et al. (2008) was identified as an outlier (11/38, 28.9%) WHO°1, and regression test showed significant funnel plot asymmetry (p = .002). After exclusion of this study, the pooled proportion was smaller (0.0%, 95%CI: 0.0%–0.1%), but the regression test was still significant (p = .015) due to the small proportions in most studies.

f Özyurt et al. (2003) was identified as an outlier (2/40, 5.0%) “other”, and regression test showed significant funnel plot asymmetry (p < .001). After exclusion of this study, the pooled proportion was similar (0.0%, 95% CI: 0.0%–1.7%), but the regression test was still significant (p = .001) due to the small proportions in most studies.

g Li et al. (2020) was identified as an influential case (5/253, 2.0%, small proportion, largest sample size) and regression test showed significant funnel plot asymmetry (p = .038). After exclusion of this study, the pooled proportion did not change considerably (8.0%, 95% CI: 6.2%–10.3%) and the regression test was no longer significant (p = .524).

h Duffau (2009) (49/51, 96.1%) and Simon et al. (2009) (68/101, 67.3%) were identified as influential cases. After exclusion of these studies, the pooled proportion did not change considerably (81.8%, 95% CI: 72.7%–88.4%).

i Per cases with epilepsy outcome.

### 3.2 Tumor growth patterns & classification attempts

Many brain tumors involving the insula also infiltrate extrainsular tissues. Tumor growth is commonly restricted to the cortices and white matter tracts constituting a developmentally older part of the brain, i.e. the so-called (para)limbic system (Yaşargil et al., 1992). Insular tumors may invade the overlying opercula, in particular the mesial aspects of parts of the temporal lobe, the fronto-orbital lobe, and also the basal ganglia and internal capsule - though involvement of the latter is less frequent than expected when considering the close anatomical relationship between the insula and basal ganglia block. The various series that form the basis of this review describe between 48.1 and 100% of tumors (pooled proportion: 81.7%, 95% CI: 72.2–88.5%; Table 2) with extrainsular tumor extensions, excluding one series devoted to gliomas growing solely within the insula (Sughrue et al., 2016). Depending on the relative amount of insular vs. extrainsular tumor infiltration, issues specific to insular surgery may be more or less relevant in an individual case. Pitskhelauri et al. therefore call into question if tumors with smaller insular extensions should be classified as insular gliomas at all (Pitskhelauri et al., 2021).

Two major classificatory attempts in the literature have aimed at systematizing the somewhat peculiar growth patterns of insular gliomas and the clinical impression that extent of resection and neurological risk seem to vary with the precise location of the tumor within the insula. Yasargil has initially proposed a classification system based on the concept of the (para)limbic system (Yaşargil et al., 1992). The Yasargil classification assigns smaller tumors more or less restricted to the insula to type 3. Type 3 A refers to tumors completely confined to the insula, while type 3 B growths infiltrate the overlying opercula. Larger growths with more prominent temporal and frontal lobe involvement are termed type 5 tumors. Type 5 B tumors infiltrate the limbic system (i.e. the hippocampus) while 5 A tumors do not. Some authors (Duffau, 2009; Duffau et al., 2009; Simon et al., 2009) use the Yasargil classification in order to help with the choice of the surgical approach (i.e. transsylvian for type 3 tumors) and also describe a correlation between extent of resection and tumor class (i.e. type 3 vs. 5). A potential correlation between survival and frontal extrainsular only was not independently confirmed (Simon et al., 2009; Wang et al., 2017). Some authors use slightly modified Yasargil classification schemes (Özyurt et al., 2003; Wang et al., 2012).

The Yasargil classification does not take into account that access to the tumor, resectability and surgical risks vary with the specific parts of the insula involved by the growth. This has prompted Sanai and Berger to develop a classification system that focuses on differential tumor growth within the insula (Sanai et al., 2010). Tumor infiltration of extrainsular tissues is not part of the classification scheme. These authors distinguish between four zones of the insula divided by a line along the Sylvian fissure and a perpendicular plane through the foramen of Monroi. Tumors involving all four zones are termed giant. Some data suggest that the Berger-Sanai classification may help to predict resection and functional outcomes. Better resection results may be obtained in more anteriorly located tumors while at the same time surgery in the antero-superior insula carries increased neurological risks (Hameed et al., 2019; Hervey-Jumper et al., 2016; Li et al., 2020; Sanai et al., 2010). Li et al. describe a modified Sanai-Berger classification and distinguish between anterior, posterior, anterior-posterior and giant tumors (Li et al., 2020). Survival seems not to vary with differential involvement of the Berger-Sanai zones (Wang et al., 2017). Of note, not all authors agree on these points (Hameed et al., 2019; Pitskhelauri et al., 2021; Przybylowski et al., 2020).

The mesial border of the tumor and its relationship to the lenticulo-striate arteries has also received considerable attention since many deficits after insular surgery seem to be related to lenticulo-striate artery compromise (see below, 3.4. Functional & epilepsy outcomes). Moshel et al. distinguish between tumors displacing or encasing the lenticulo-striate arteries (as assessed by catheter angiography and MRI) and describe better resection and functional outcomes in the displacement only group (Moshel et al., 2008). Rao and co-workers use 3D TOF (time of flight) and CISS (coronal constructive interference in steady state) MR sequences for lenticulo-striate arteries imaging and detail similar results (Rao et al., 2018). Lack of lenticulo-striate arteries involvement and clear (including mesial) tumor boundaries were proposed as indicators to identify candidates for aggressive resections (Kawaguchi et al., 2014). Wang et al. classify insular tumors according to their infiltrative behavior with respect to the putamen as seen on MR imaging (Wang et al., 2017). Putaminal involvement was found to correlate with a lesser extent of resection and worse survival.

Pitskhelauri and co-workers distinguish between purely insular tumors without basal ganglia involvement, tumors with >50% insular involvement but temporal or frontal lobe extensions, tumors with >50% insular growth and putaminal involvement, and tumor with >50% extrainsular growth. These authors found the best resection outcomes in purely insular and in >50% extrainsular tumors, and deficits only in the latter two groups (Pitskhelauri et al., 2021).

### 3.3 Extent of resection & residual tumor

Complete resections of insular tumors are difficult to achieve. Hence, many authors do not report 100% resections but rather GTR rates or >90% (95%) resection outcomes. Some authors report residual tumor volumes rather than degree of resection (Duffau, 2009; Przybylowski et al., 2020). For analytical purposes we therefore evaluated the percentage of cases in the respective series in the “best” or >90% resection category as revealed by postoperative routine neuroimaging. This figure varied between 14.1% and 69.6% in series including >100 cases. Overall, GTR and >90% resections were reported in 53.7% (pooled proportion; 95% CI: 43.6–63.5%; Table 2).

Residual tumor after surgery is not randomly distributed. Residual tumor is most often reported mesially, i.e. close to the lenticulo-striate arteries, in the anterior perforate substance, in projection on the internal capsule and basal ganglia, but also in the posterior insula (Hameed et al., 2019; Hervey-Jumper et al., 2016; Kawaguchi et al., 2014; Leroy et al., 2021; Moshel et al., 2008; Pitskhelauri et al., 2021; Rao et al., 2018; Rossi et al., 2021; Sanai et al., 2010; Sughrue et al., 2017; Wang et al., 2012; Wang et al., 2017). Hameed et al. describe tumor remnants in the region of the superior periinsular sulcus where the distance to the motor tract is very small (Hameed et al., 2019). As a possible corollary, Kawaguchi and co-workers associate better resections with an intact superior extremity of the central insular sulcus (Kawaguchi et al., 2014). More extensive insular growth (“giant” tumors) and extrainsular tumor growth have also been associated with worse resection outcomes (Hameed et al., 2019; Hervey-Jumper et al., 2016; Li et al., 2020; Pitskhelauri et al., 2021; Sanai et al., 2010).

### 3.4 Functional & epilepsy outcomes

**Fig. 2:**
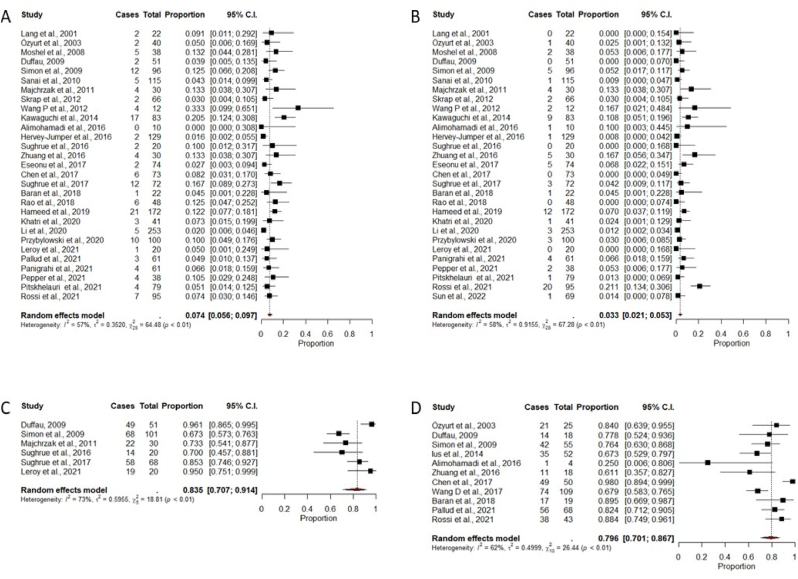
Forest plots for functional outcomes.

Neurological deficits are not uncommon, and many author specifically detail motor and speech/language deficit rates. Permanent motor impairment is reported in 0–33.3% (pooled proportion: 7.4%, 95% CI: 5.6–9.7%) of cases, and aphasia or other language/speech deficits in 0–13.3% (pooled proportion; per all surgical cases, i.e. most authors do not report handedness or other measures of hemispherical dominance: 3.3%, 95% CI: 2.1–5.3%; Table 2 & Fig. 2A and B). These figures vary with the size of the respective series with lesser deficits seen in larger series, however, differences were not statistically significant (pooled proportions; ≤50 vs. > 50 cases, motor deficits: 9.5%, 95% CI: 6.1%–14.5% vs. 6.5 %, 95% CI: 4.6%–9.1%, p = .168 & language/speech impairment: 4.0%, 95% CI: 1.9%–8.1% vs. 3.1%, 95% CI: 1.7%–5.3%, p = .568). The rate of temporary deficits is high and several publications describe neurological improvement over time. Specifically, early motor and language deficit rates in series describing >50 surgeries varied between 8.1 - 37.5% and 4.3–50.1% (Duffau, 2009; Eseonu et al., 2017; Hameed et al., 2019; Hervey-Jumper et al., 2016; Kawaguchi et al., 2014; Lang et al., 2001; Leroy et al., 2021; Li et al., 2020; Majchrzak et al., 2011; Moshel et al., 2008; Özyurt et al., 2003; Pallud et al., 2021; Pepper et al., 2021; Pitskhelauri et al., 2021; Przybylowski et al., 2020; Rao et al., 2018; Rossi et al., 2021; Sanai et al., 2010; Simon et al., 2009; Skrap et al., 2012; Sughrue et al., 2017; Sughrue et al., 2016; Sun et al., 2022; Zhuang et al., 2016). The majority of neurological complications seems to be caused by ischemia due to small vessel compromise, i.e. lenticulo-striate artery and M3/M2 perforator infarcts (Chen et al., 2017; Duffau, 2009; Hameed et al., 2019; Kawaguchi et al., 2014; Li et al., 2020; Moshel et al., 2008; Pallud et al., 2021; Pitskhelauri et al., 2021; Rao et al., 2018; Simon et al., 2009; Skrap et al., 2012; Sughrue et al., 2017; Zhuang et al., 2016). Several authors report on postoperative functional outcomes using the KPI scale. The overall percentage of cases with a postoperative KPI 80–100 in the cohorts reviewed varies between 67.3 and 96.1% (pooled proportion: 83.5%, 95% CI: 70.7–91.4%; Table 2 & Fig. 2C).

Several authors comment on tumor associated epilepsy and seizure outcomes. Seizure incidence in the various series may primarily reflects the histological composition of the respective cohort. Excluding series describing only patients with epilepsy, between 35.1 and 98.0% of patients report preoperative seizures (Table 1A). Epilepsy outcomes after insular surgery appear to be quite good. Eleven series describe 461 patients with tumor-associated epilepsy and 61.1–98.0% seizure-free rates following surgery in cohorts with >10 cases with epilepsy (pooled proportion: 79.6%, 95% CI: 70.1–86.7%, Table 2 & Fig. 2D). Two papers describe a correlation between extent of resection and epilepsy control (Ius et al., 2014; Wang et al., 2018).

### 3.5 Choice of the surgical approach

Insular tumors can be accessed through a transsylvian route, or – alternatively – through transcortical trajectories. The transsylvian route was pioneered by Yasargil and co-workers (Yaşargil et al., 1992). Transtemporal and transfrontal approaches, i.e. operating insular tumors through the temporal stem following a temporal lobe resection or entering the insula through a frontal or fronto-basal corticotomy (Simon et al., 2009; Sun et al., 2022, 2023), were described later on reflecting growing experience with surgery for large tumors with extrainsular extensions. Transsylvian surgery involves often significant manipulation of the vasculature of the Sylvian fissure with an attendant risk for vascular injuries (Lang et al., 2001; Przybylowski et al., 2021). Transopercular approaches were developed in part to overcome this problem. Advances in intraoperative cortical mapping and awake surgery allowed for insular tumor removal following resection of potentially eloquent (fronto)-opercular tissues (Duffau, 2009; Sanai et al., 2010). The specifics of the individual tumor growth pattern with respect to extrainsular tumor extensions and involvement of different parts of the insula of course play a role when choosing the surgical approach in many series. Many authors routinely approach small tumors restricted to the insula through the transsylvian route (Duffau, 2009; Hameed et al., 2019; Lang et al., 2001; Panigrahi et al., 2021; Pitskhelauri et al., 2021; Zhuang et al., 2016).

Panigrahi et al. and Przybylowski and co-workers compare clinical results after transsylvian vs. transcortical surgery (Panigrahi et al., 2021; Przybylowski et al., 2020) and report somewhat more complications following transsylvian surgery. For analytical purposes we categorized the studies included in this review according to their policy with respect to surgical approaches, i.e. we distinguished between studies relying exclusively on either transssylvian or transcortical surgery, or utilized both approaches. A meta-analysis indeed showed somewhat higher rates of motor but not language deficits following transsylvian surgery (Table 3). The proportion of tumor with extrainsular growth was lower in the series relying on transsylvian surgery only.

**Table 3:** Surgical approach vs. functional and resection outcomes. Studies were assigned to the respective categories depending on if patients were operated exclusively through a transsylvian approach, if no transsylvian surgeries were performed, or if both transsylvian and transcortical/-opercular approaches were used.

	Transsylvian		Transsylvian & transopercular/-cortical		No transsylvian		All		Test for subgroup differences	
	Patients (studies)	Pooled proportion (95% CI)	Patients (studies)	Pooled proportion (95% CI)	Patients (studies)	Pooled proportion (95% CI)	Patients (studies)	Pooled proportion (95% CI)	χ^2^ (2)	p
Extrainsular growth^a^	221 (6)	66.0% (47.5%–80.7%)	388 (7)	90.4% (81.4%–95.2%)	521 (4)	86.1% (71.4%–93.9%)	1130 (18)	83.3% (73.6%–90.0%)	8.75	0.013
EOR >90%^b^	289 (8)	56.8% (35.6%–75.9%)	522 (9)	57.4% (37.8%–74.9%)	1170 (10)	59.6% (40.8%–75.8%)	1981 (27)	58.1% (46.5%–68.8%)	0.04	0.979
Postop. motor deficit^c^	289 (8)	10.2% (6.4%–15.9%)	590 (9)	8.6% (5.8%–12.5%)	941 (9)	4.4% (2.9%–6.9%)c	1820 (26)	7.0% (5.3%–9.2%)	7.84	0.020
Postop. speech deficit^d^	289 (8)	2.8% (1.0%–7.3%)	590 (9)	3.1% (1.4%–6.9%)	949 (9)	3.0% (1.3%–6.6%)d	1828 (24)	3.0% (1.8%–4.9%)	0.04	0.982
Postop. KPI 80–100^e^^,^^f^	50 (3)	84.5% (70.5%–92.6%)	220 (2)	72.4% (47.8%–88.2%)	20 (1)	95.5% (66.6%–99.6%)	290 (6)	83.5% (70.7%–91.4%)	2.87	0.238
Seizure outcome (per cases with epilepsy)^e^	25 (1)	84.7% (53.9%–96.3%)	141 (4)	82.3% (69.0%–90.7%)	130 (3)	86.8% (73.8%–93.9%)	296 (8)	84.5% (76.0%–90.4%)	0.37	0.830

EOR: extent of resection.

95% CI: 95% confidence interval.

KPI: Karnofsky performance index.

a Exclusion of Sughrue et al. (2016) because patients with extrainsular growth were not included. Outlier/influential study: Zhuang et al. (2016); 17/31 (56.7%), transsylvian & transcortical approaches, not removed.

b Cases with a gross total resection or EOR (extent of resection) > 90 (95)%.

c Outlier/influential study: Hameed et al. (2019); 21/172 (12.2%), no transsylvian approaches, not removed.

d Outlier/influential study: Rossi et al. (2021); 20/95 (21.1%), no transsylvian approaches, not removed.

e Small number of studies.

f Outlier/influential studies: Duffau (2009); 49/51 (96.1%) & Simon et al. (2009) 68/101 (67.3%), both transsylvian & transcortical approaches, not removed.

### 3.6 Awake craniotomy

Surgical adjuncts were widely used. Twenty-six assessable series report the routine use of one or more intraoperative functional mapping and/or monitoring techniques (including awake craniotomy in N = 19 cohorts). Four studies describe the utilization of advanced (intraoperative) imaging including neuronavigation ultrasound and intraoperative MR imaging, but no monitoring/mapping paradigm (Chen et al., 2017; Sughrue et al., 2017; Sughrue et al., 2016; Wang et al., 2012). The cases reported by Leroy et al. were operated under general anesthesia (and with intraoperative MRI). However, all tumors were located in the non-dominant hemisphere and the authors state that they prefer awake surgery for gliomas of the dominant hemisphere (Leroy et al., 2021). Of note, descriptions of the techniques and paradigms used varied between series and were not reported on a case-by-cases basis, and during the accumulation of the respective cohorts fairly often several monitoring/mapping strategies were utilized which precluded many comparative analyses.

Together these findings prompted us to study the potential role of awake surgery for insular tumors. We found somewhat better motor, but also significantly worse resection outcomes to be associated with the use of awake craniotomies (Table 4A). Since the great majority of cases in our analysis had surgery with the help of some mapping or monitoring paradigm we felt that any analysis including cases operated without such adjuncts would probably suffer from much bias. We therefore repeated our analysis after exclusion of these latter cohorts. This resulted in generally similar findings, however, the statistical significance of the association between lesser degrees of resection and awake surgery was lost (Table 4B). Awake surgery was also associated with better KPI outcomes, however this was based on just one study (Tab. 4 A, Tab. 4 BA and B).

**Tab. 4 A:** Pooled functional and resection outcomes & use of awake craniotomy (all series).

	Yes		No		All		Test for subgroup differences	
	Patients (studies)	Pooled proportion (95% CI)	Patients (studies)	Pooled proportion (95% CI)	Patients (studies)	Pooled proportion (95% CI)	χ^2^ (1)	p
EOR >90%^a^	1286 (16)	42.9% (30.3%–56.4%)	813 (14)	67.1% (52.9%–78.8%)	2099 (30)	54.4% (43.2%–65.2%)	5.86	0.016
Postop. motor deficit^b^	1226 (17)	6.8% (4.7%–9.6%)	685 (11)	8.9% (5.7%–13.7%)	1911 (28)	7.5% (5.7%–9.9%)	0.92	0.338
Postop speech deficit	754 (12)	3.3% (1.6%–6.6%)	1165 (16)	3.5% (1.9%–6.3%)	1919 (28)	3.4% (2.1%–5.4%)	0.02	0.893
Postop. KPI 80–100^c^^,^^d^	51 (1)	96.3% (83.5%–99.2%)	239 (5)	78.0% (67.9%–85.6%)	290 (6)	83.5% (70.7%–91.4%)	5.23	0.022
Seizure outcome (per cases with epilepsy)^c^^,^^e^	166 (5)	82.0% (65.5%–91.6%)	109 (3)	82.5% (58.1%–94.1%)	275 (8)	82.2% (69.4%–90.4%)	<0.01	0.968

EOR: extent of resection.

95% CI: 95% confidence interval.

KPI: Karnofsky performance index.

a Cases with a gross total resection or EOR (extent of resection) > 90 (95)%.

b Influential study: Li et al. (2020); 5/253 (2.0%), small proportion, largest sample size, awake: no, not removed.

c Small number of studies.

d Outlier/influential studies: Simon et al. (2009); 68/101 (67.3%), awake: no & Sughrue et al. (2017); 58/68 (85.3%), awake: no, both not removed.

e Outlier/influential study: Chen et al. (2017); 49/50 (98.0%), awake: no, not removed.

**Tab. 4 B:** Pooled functional and resection outcomes & use of awake craniotomy (only series employing functional mapping/monitoring strategies).

	Yes		No			All		Test for subgroup differences	
	Patients (studies)	Pooled proportion (95% CI)	Patients (studies)		Pooled proportion (95% CI)	Patients (studies)	Pooled proportion (95% CI)	χ^2^ (1)	p
EOR >90%^a^	1286 (16)	42.9% (30.4%–56.3%)	616 (9)		59.1% (41.1%–75.0%)	1902 (25)	48.7% (37.5%–60.0%)	2.01	0.156
Postop. motor deficit^b^	1095 (15)	5.9% (4.1%–8.4%)	488 (6)		6.9% (4.0%–11.9%)	1583 (21)	6.1% (4.5%–8.3%)	0.25	0.616
Postop speech deficit	1165 (16)	3.5% (1.9%–6.3%)	557 (7)		4.1% (1.7%–9.2%)	1722 (23)	3.7% (2.3%–5.9%)	0.08	0.774
Postop. KPI 80–100^c^	51 (1)	96.1% (85.6%–99.1%)	131 (2)		68.7% (60.3%–76.1%)	182 (3)	82.5% (58.9%–94.0%)	10.47	0.001
Seizure outcome (per cases with epilepsy)^c^	166 (5)	81.9% (75.3%–87.1%)	59 (2)		72.9% (60.2%–82.7%)	225 (7)	79.2% (71.7%–85.2%)	2.16	0.142

EOR: extent of resection.

95% CI: 95% confidence interval.

KPI: Karnofsky performance index.

a Cases with a gross total resection or EOR (extent of resection) > 90 (95)%.

b Influential study: Li et al. (2020); 5/253 (2.0%), small proportion, largest sample size, awake: no, not removed.

c Small number of studies.

### 3.7 Patient survival

Survival outcomes vary somewhat between the series reviewed for this paper. 1 year overall survival for glioblastomas was 20–96%. 5 yr. survival varied between 35 and 74% for anaplastic (WHO/CNS grade 3) and 57–93% for "low grade“ (i.e. WHO grade 2) tumors (Table 1B).

### 3.8 Insular glioblastomas

Some authors describe worse functional outcomes in patients with glioblastomas and therefore recommend a somewhat cautious approach in such cases (Lang et al., 2001; Przybylowski et al., 2020; Simon et al., 2009; Singh et al., 2023; Sughrue et al., 2017). While many studies report surgeries with insular glioblastomas, only very few detail functional outcomes according to histology. The paper by Singh et al. specifically investigates surgery for insular glioblastomas (Singh et al., 2023). A meta-analysis of the postoperative KPI provided some evidence for an association between a worse postoperative KPI and glioblastoma histology (postoperative KPI 80–100: pooled proportion 0.150, 95% CI 0.069–0.325), however, was based on 4 studies with 187 patients (62 with glioblastoma) only (Leroy et al., 2021; Simon et al., 2009; Sughrue et al., 2017; Sughrue et al., 2016). Five studies (132 patients, 24 with glioblastomas) provided motor outcomes in patients with glioblastomas vs. all other histologies (motor deficit: pooled proportion 2.310, 95% CI 0.151–35.438) (Lang et al., 2001; Leroy et al., 2021; Majchrzak et al., 2011; Özyurt et al., 2003; Sughrue et al., 2017). Four studies (Lang et al., 2001; Leroy et al., 2021; Özyurt et al., 2003; Sughrue et al., 2017) report overall 0/21 speech deficits in glioblastoma cases vs. 1/81 in other histologies.

### 3.9 Molecular findings

Several studies (Eseonu et al., 2017; Hameed et al., 2019; Hervey-Jumper et al., 2016; Pallud et al., 2021; Pepper et al., 2021; Pitskhelauri et al., 2021; Rossi et al., 2021; Tang et al., 2016; Wang et al., 2017) describe molecular alterations in addition to or as part of the description of the histological findings of the tumors reported. Most commonly, the respective authors study IDH1 mutations and 1p/19q co-deletions which are included in the current WHO classification as diagnostic markers, i.e. these analyses investigate potential differences between oligodendrogliomas and astrocytomas, and the clinical characteristics of IDH mutant gliomas (“lower grade” gliomas) rather than molecular biomarkers (Louis et al., 2021). Both alterations were found to correlate with better progression-free and overall survival in some series (Eseonu et al., 2017; Hameed et al., 2019; Pallud et al., 2021; Tang et al., 2016; Wang et al., 2017). No correlations were seen with deficit rates (Rossi et al., 2021) or seizure outcomes (Pepper et al., 2021; Wang et al., 2018). Some authors investigated potential associations between molecular findings and tumor growth patterns and report inconclusive or conflicting data (Hameed et al., 2019; Pitskhelauri et al., 2021; Tang et al., 2016; Wang et al., 2017).

## 4 Discussion

The pertinent literature contains several systematic reviews and a few meta-analyses investigating the published experience with insular glioma surgery (Di Carlo et al., 2020; Lu et al., 2019; Papadopoulou & Kumar, 2024; Zhang et al., 2022). Some narrative reviews have also been published in recent years (Duffau, 2018; Hervey-Jumper & Berger, 2019; Murrone et al., 2019; Przybylowski et al., 2021; Renfrow et al., 2023). Limitations of the literature may include the somewhat limited scope of many publications. There may also be a certain bias related to the exclusion of otherwise significant publications because they do not contain the prespecified data. The present analysis was therefore conducted in order to provide a more systematic and complete overview of the published experience with insular tumor surgeries including resection but also functional outcome figures.

### 4.1 Insular glioma surgery outcomes

Central findings of our review include pooled 6.8% postoperative motor and 3.6% speech deficit rates, and a 83.5% chance of a postoperative KPI 80–100. The 2019 meta-analysis by Lu et al. (2019) reports slightly lower figures, i.e. pooled incidences of new permanent motor deficits in 4% (95% CI: 2–7%) and language deficits in 2% (95% CI: 0–4%). In view of the eloquent location of insular tumors these figures compare not unfavorably with deficit rates and outcomes reported in various glioma series in the literature. E.g. Awad et al. report a mean postoperative KPI of 80.0 ± 16.6 in a cohort of 330 glioblastoma patients (Awad et al., 2017). The paper by Svenjeby and co-workers details overall 4.0% severe (any: 21.8%) motor and 3.0% severe (any: 15.8%) language deficits in a series of 202 IDH1 mutant “lower” grade gliomas (Svenjeby et al., 2022). Zhang et al. describe 734 cases with gliomas of all WHO grades and found a 10.6% late motor and 7.2% late language deficit rate (Zhang et al., 2018).

The pooled >90% resection rate was 60.8% (95% CI: 50.7–69.9%) which actually compares reasonably with glioma cohorts not selected for tumor location. Gross total or complete resection rates in the range of 17.0–64.0% have been reported in more recent publications (Gousias et al., 2014; Hervey-Jumper et al., 2023; Obara et al., 2020; Stupp et al., 2017; Svenjeby et al., 2022). Collectively, these figures suggest that specialized institutions may be able to achieve quite acceptable deficit and resection rates for tumors in difficult locations. In support of this view we found somewhat lesser deficit rates in larger (>50 cases) series even though findings were not statistically significant. Series size may be considered a proxy for specialization. It might well be that the risk and complication rates of everyday clinical practice are often underestimated, while many have a false negative impression of surgical results for difficult tumors based on a relative lack of experience with the management of these lesions. This may be an important argument in favor of surgery for insular glioma (and possibly other tumors in eloquent locations as well).

Resectability and deficit rates vary significantly with tumor location and growth pattern. Incomplete resections appear to reflect accessibility issues (posterior insula, overall tumor size and extension) (Hameed et al., 2019; Hervey-Jumper et al., 2016; Li et al., 2020; Özyurt et al., 2003; Pitskhelauri et al., 2021; Sanai et al., 2010) and in particular functional concerns. There seems to be a general consensus that the mesial border of the tumor and its relationship to the lenticulostriate arteries as well as the long M2/3 perforators play a major role in this regard (Chen et al., 2017; Duffau, 2009; Hameed et al., 2019; Hervey-Jumper et al., 2016; Kawaguchi et al., 2014; Leroy et al., 2021; Li et al., 2020; Moshel et al., 2008; Özyurt et al., 2003; Pallud et al., 2021; Pitskhelauri et al., 2021; Rao et al., 2018; Rossi et al., 2021; Sanai et al., 2010; Simon et al., 2009; Skrap et al., 2012; Sughrue et al., 2017; Wang et al., 2012; Wang et al., 2017; Zhuang et al., 2016).

Our analysis also highlights important limitations of the current literature on neurological and functional outcomes following brain tumor surgery. Neurological deficits were not reported in a standardized manner and often reported for different time points or without mentioning the time point at all. In contrast to others (Di Carlo et al., 2020; Lu et al., 2019) we therefore decided against studying temporary deficits – which frequently occur following insular tumor surgery (Duffau, 2009; Eseonu et al., 2017; Hameed et al., 2019; Hervey-Jumper et al., 2016; Kawaguchi et al., 2014; Lang et al., 2001; Leroy et al., 2021; Li et al., 2020; Majchrzak et al., 2011; Moshel et al., 2008; Özyurt et al., 2003; Pallud et al., 2021; Pepper et al., 2021; Pitskhelauri et al., 2021; Przybylowski et al., 2020; Rao et al., 2018; Rossi et al., 2021; Sanai et al., 2010; Simon et al., 2009; Skrap et al., 2012; Sughrue et al., 2017; Sughrue et al., 2016; Sun et al., 2022; Zhuang et al., 2016).

Many patients with insular tumors suffer from epilepsy and seizures. Data reporting varies a lot between studies, and many studies do not use typical epilepsy outcome scales such as the Engel or ILAE classification (Alimohamadi et al., 2016; Duffau, 2009; Özyurt et al., 2003; Pallud et al., 2021; Zhuang et al., 2016). Our results suggest surprisingly good epilepsy outcomes following insular surgery, i.e. a 79.6% rate of becoming seizure-free. For comparison, Ollila and Roivainen report a 57.6% seizure-free rate in a series of 123 glioma cases (not selected for location) which includes 70.7% patients presenting with tumor-associated epilepsy (Ollila & Roivainen, 2023). 60.0% of the 382 patients with WHO grade 2 gliomas studied by Hervey-Jumper and co-workers were seizure-free after surgery (Hervey-Jumper et al., 2023). This is a noteworthy finding since truly complete insular tumor resections are rarely possible and from an epilepsy surgery perspective complete lesionectomies (and usually even more extensive resections) are necessary for epilepsy control. On the other hand, there are some data to suggest that even incomplete resections may be helpful which somewhat strengthens the view that insular surgery also aims at epilepsy control despite the often limited resectability of the tumors. Zhang et al. have recently published a meta-analyses of epilepsy outcomes following insular surgery. These authors also report a very similar pooled seizure freedom rate at 1 year of 78% (Zhang et al., 2022). In addition, they describe an optimal threshold for seizure freedom of ca. 80% confirming earlier work by Xu et al. who studied the same question in a low-grade glioma cohort not selected for tumor location (Xu et al., 2018).

Within the limits of a systematic review we obtained no evidence that patients with insular gliomas might live longer than patients with the same histologies in other brain locations (Gousias et al., 2014; Hervey-Jumper et al., 2023; Lassman et al., 2022; Obara et al., 2020; Stupp et al., 2017; van den Bent et al., 2021).

### 4.2 Choice of the surgical approach

Insular gliomas can be approached either via a transsylvian or transcortical trajectories. The current literature contains two papers which specifically investigate this issue and indeed report slightly more complications and deficits following transsylvian surgery (Panigrahi et al., 2021; Przybylowski et al., 2020) which prompted us to attempt a meta-analysis of the available literature. Since individual cases data were not available and most studies did not detail outcome differences between tumor subsets characterized by the surgical approach used, we rather distinguished between studies reporting exclusively transsylvian or transcortical surgeries, or both. Our analysis indeed provides some evidence in favor of transcortical surgeries, i.e. higher motor deficit rates were seen in the series relying on transsylvian surgery in at least some of the cases reported. It should be noted, however, that the choice of the surgical approach is not completely arbitrary, but rather depends significantly on the specifics of the tumor growth pattern in an individual case.

### 4.3 Intraoperative mapping/monitoring & awake surgery

The great majority of assessable studies (26 of 31; three papers did not provide the pertinent information) reported the routine use of some kind of intraoperative mapping/monitoring paradigm, i.e. there seems to be almost a consensus to incorporate some kind of functional diagnostics into one's surgical strategy. However, the specifics of the respective institutional paradigms varied significantly. We studied the role of awake surgery. Functional outcomes were generally better and resection outcomes were worse in the cohorts which reported use of awake surgery. On first glance, the latter finding is somewhat unexpected since most of the literature investigating monitoring and awake surgery as tools to improve neurological outcomes also report better resection rates (De Witt Hamer et al., 2012; Gerritsen et al., 2022). Indeed, Pallud et al. investigated their institutional experience with asleep vs. awake surgery for insular gliomas and found better resection, epilepsy and 3 months KPI outcomes in the asleep group (Pallud et al., 2021).

However, these latter studies usually compare surgeries which incorporate a monitoring/mapping strategy to operations which (at least to a large percentage) do not. As mentioned above, there is almost a consensus that insular surgery requires some kind of intraoperative functional diagnostics. Hence, our findings may reflect in part the difference between including awake surgery into one's monitoring/mapping strategy vs. not, and therefore suggest that operating insular tumors awake rather than asleep with e.g. MEP monitoring may result in slightly better functional but also worse resection outcomes. Importantly, the differences observed were not statistically significant (after exclusion of series not using a monitoring/mapping paradigm).

Awake surgery is most often performed when preservation of language functions is the major concern. One potential explanation for our findings may therefore be that causes of language deficits following surgery differ between insular and lobar gliomas. Deficits after insular surgery are usually related to small perforator compromise resulting in distant infarctions. This may be somewhat difficult to prevent using an electrostimulation paradigm during awake surgery which conceptually relies heavily on keeping a safe distance from the structure (rather than the vasculature) that needs to be preserved.

Duffau compared awake surgery for right-sided insular gliomas to asleep surgery with neuromonitoring based on his experience in two consecutive (1997–2009: asleep, 2009–2020: awake) experiences. He found lesser deficits but similar resection outcomes in the more recently operated patients (Duffau, 2022). Di Carlo et al. recently addressed the same question in their meta-analysis but followed a somewhat different approach (Di Carlo et al., 2020). These authors analyzed overall deficit rates despite potential underreporting of non-motor, non-language deficits and attempted to extract individual case data (or at least deficit rates in cases undergoing awake surgery as compared to surgery under general anesthesia). This resulted in excluding many (and in particular the larger) studies reviewed in our investigation. Similar to our findings their results indicate lesser deficits in awake craniotomy patients when compared to cases undergoing surgery under general anesthesia.

### 4.4 Insular glioblastoma surgery

Patients with glioblastomas of the insula are more often not considered surgical candidates than cases with other presumed histologies. Indeed, the 16.2% (95% CI: 8.7%–28.1%) proportion of glioblastomas in the series reviewed for this paper is relatively low when compared to unselected glioma surgery cohorts (cf. Zhang et al., 2018: 39.2%). Interestingly, the literature reviewed was found to contain surprisingly few pertinent data in support of this somewhat cautious attitude, i.e. functional outcome information in cases with glioblastoma vs. other histologies. Given the relative frequency of glioblastomas and the commonly voiced concerns with respect to resective surgery in such cases, this issue seems to warrant more investigation and a better database for decision making.

### 4.5 Growth pattern & classification issues

The majority of studies included in this review utilize Yasargil's classification scheme (Yaşargil et al., 1992) and/or the Berger-Sanai paradigm (Hervey-Jumper et al., 2016; Sanai et al., 2010). We failed to identify robust data indicating that the use of these classifications might help substantially with outcome prediction and surgical decision making. It would appear that this is another question that may deserve more scientific attention. The specifics of the mesial border of the tumor i.e. perforator and basal ganglia involvement clearly impact on resectability and deficit rates which would argue that this aspect of tumor growth would have to be included in a future robust classification scheme (Pitskhelauri et al., 2021; Wang et al., 2017). In the meantime, it may be most appropriate to report insular glioma series using both the Yasragil and the Berger-Sanai classification scheme, and an item that describes the mesial border of the tumors.

Frontal and temporal lobe infiltration is another important issue. Extrainsular growth may determine the surgical approach. The present study details a 83.3% (95% CI: 73.6%–90.0%) rate of tumors with extrainsular extensions, i.e. most “insular” glioma resections involve extrainsular tissues. Since degree of resection probably matters regardless of the location of residual tumor, our data suggest more focus on removing extrainsular rather than insular tumor in order to enhance resection outcomes and help with deficit avoidance.

### 4.6 Limitations

The present study suffers from significant limitations. Data reporting was not standardized, and we often had to accept somewhat crude outcome categories for further analyses. This clearly impacted negatively on our ability to draw robust conclusions. Individual case data was not available for most and particularly the larger studies. Tests for heterogeneity for the items investigated were usually significantly positive indicating that surgical indications vary considerable between centers as well as publication bias.

## 5 Conclusions

Nevertheless our study analyses >2200 published patients undergoing insular glioma surgery. This allows to report fairly robust pooled outcome figures such as 6.8% risk of postoperative motor and a 3.6% risk of speech deficits, a 83.5% chance of a postoperative KPI 80–100, a 60.8% chance for a >90% resection and a 79.6% epilepsy control rate. Including a functional monitoring/mapping paradigm (which may include awake craniotomies) into one's surgical strategy seems mandatory. We found some evidence that transcortical approaches may carry a lesser rate of (motor) deficits than transsylvian surgeries. Questions and issues that warrant more attention include surgery for insular glioblastomas and how to classify the various growth patterns. More standardized reporting of functional and resection outcomes as well as other items will be necessary in order to arrive at more robust answers to the controversies addressed in the review.

## Funding

This research did not receive any specific grant from funding agencies in the public, commercial, or not-for-profit sectors.

## Declaration of competing interest

The authors declare that they have no known competing financial interests or personal relationships that could have appeared to influence the work reported in this paper.
