# Supplementary material for: Surgical treatment for insular gliomas. A systematic review and meta-analysis on behalf of the EANS neuro-oncology section
Source: Brain Spine. 2024 May 15;4:102828. doi: 10.1016/j.bas.2024.102828 (PMC11163152; doi:10.1016/j.bas.2024.102828)
Supplement: Multimedia component 1 [file mmc1.docx]

Supplemental Table 1: Search syntax

| **Database** | **N** | **Syntax** |
| --- | --- | --- |
| Embase | 460 | ('insular glioma'/de OR ('insula'/exp AND ('glioma'/de OR 'brain tumor'/de)) OR (((insula OR insular) NEAR/3 (glioma* OR tumor* OR tumour* OR neoplas* OR cancer*)) NOT ((insular) NEAR/3 (thyroid* OR pancrea*))):ab,ti,kw) **AND** ('surgery'/exp OR 'cancer surgery'/exp OR 'brain surgery'/exp OR 'surgical technique'/exp OR 'glioma'/'surgery'/de OR 'intraoperative period'/de OR (surger* OR surgical OR operat* OR resect* OR microsurg* OR neurosurg* OR intraoperativ*):ab,ti,kw) AND [ENGLISH]/lim NOT ((animal/exp OR animal*:de OR nonhuman/de) NOT ('human'/exp)) |
| Medline | 196 | ((Insular Cortex/ AND (Glioma/ OR Brain Neoplasms/)) OR (((insula OR insular) ADJ3 (glioma* OR tumor* OR tumour* OR neoplas*)) NOT ((insular) ADJ3 (thyroid* OR pancrea*))).ab,ti,kf.) **AND** (exp Specialties, Surgical/ OR exp Surgical Procedures, Operative/ OR surgery.fs. OR glioma/su OR Intraoperative Period/ OR (surger* OR surgical OR operat* OR resect* OR microsurg* OR neurosurg* OR intraoperativ*).ab,ti,kf.) AND english.la. NOT (exp Animals/ NOT Humans/) |
| Cochrane | 4 | ((((insula OR insular) NEAR/3 (glioma* OR tumor* OR tumour* OR neoplas* OR cancer*)) NOT ((insular) NEAR/3 (thyroid* OR pancrea*))):ab,ti) **AND** ((surger* OR surgical OR operat* OR resect* OR microsurg* OR neurosurg* OR intraoperativ*):ab,ti) |
| Web of Science | 201 | TS=(((((insula OR insular) NEAR/2 (glioma* OR tumor* OR tumour* OR neoplas* OR cancer*)) NOT ((insular) NEAR/2 (thyroid* OR pancrea*)))) **AND** ((surger* OR surgical OR operat* OR resect* OR microsurg* OR neurosurg* OR intraoperativ*)) NOT ((animal* OR rat OR rats OR mouse OR mice OR murine OR dog OR dogs OR canine OR cat OR cats OR feline OR rabbit OR cow OR cows OR bovine OR rodent* OR sheep OR ovine OR pig OR swine OR porcine OR veterinar* OR chick* OR zebrafish* OR baboon* OR nonhuman* OR primate* OR cattle* OR goose OR geese OR duck OR macaque* OR avian* OR bird* OR fish*) NOT (human* OR patient* OR women OR woman OR men OR man))) AND LA=(English) |
| Google Scholar | 100 | "insula\|insular glioma\|gliomas" surgery\|surgical\|operation\|resected\|resection\|microsurgy\|neurosurgy\|intraoperative |
